# Clinical Pathway and Monthly Feedback Improve Adherence to Antibiotic Guideline Recommendations for Community-Acquired Pneumonia

**DOI:** 10.1371/journal.pone.0159467

**Published:** 2016-07-25

**Authors:** Maher Almatar, Gregory M. Peterson, Angus Thompson, Duncan McKenzie, Tara Anderson, Syed Tabish R. Zaidi

**Affiliations:** 1 Pharmacy, School of Medicine, University of Tasmania, Hobart, Tasmania, Australia; 2 Royal Hobart Hospital, Hobart, Tasmania, Australia; University of California San Diego, UNITED STATES

## Abstract

**Background:**

Compliance with community-acquired pneumonia (CAP) guidelines remains poor despite a substantial body of evidence indicating that guideline-concordant care improves patient outcomes. The aim of this study was to compare the relative effectiveness of a general educational and a targeted emergency department intervention on improving physicians’ concordance with CAP guidelines.

**Methods:**

Two distinct interventions were implemented over specific time periods. The first intervention was educational, focusing on the development of local CAP guidelines and their dissemination through hospital-wide educational programmes. The second intervention was a targeted one for the emergency department, where a clinical pathway for the initial management of CAP patients was introduced, followed by monthly feedback to the emergency department (ED) physicians about concordance rates with the guidelines. Data on the concordance rate to CAP guidelines was collected from a retrospective chart review.

**Results:**

A total of 398 eligible patient records were reviewed to measure concordance to CAP guidelines over the study period. Concordance rates during the baseline and educational intervention periods were similar (28.1% vs. 31.2%; p > 0.05). Significantly more patients were treated in accordance with the CAP guidelines after the ED focused intervention when compared to the baseline (61.5% vs. 28.1%; p < 0.05) or educational period (61.5% vs. 31.2%; p < 0.05).

**Conclusions:**

A targeted intervention with a CAP clinical pathway and monthly feedback was a successful strategy to increase adherence to empirical antibiotic recommendations in CAP guidelines.

## Introduction

Community-acquired pneumonia (CAP) is a major cause of hospitalisation and death, both in Australia and around the world[1, 2]. The inpatient mortality rate for patients hospitalised with CAP was as high as 23% in one US epidemiological study[1]. Pneumonia was responsible for more than 2500 deaths in Australia for the year 2012 [2]; this represents almost 2% of all deaths. A number of practice guidelines exist to facilitate the prompt and effective treatment of CAP[3–5]. Concordance to CAP guidelines has been associated with an improved process of care [6, 7] and leads to better clinical outcomes [3]. Nevertheless, physicians’ concordance to practice guidelines for CAP has been shown to be poor within Australian hospitals [8, 9].

Several barriers contributing to the poor concordance to CAP guidelines have been identified [10, 11]. Concerns about the lack of appropriate antibiotic cover, availability of conflicting practice-guidelines, poor guideline integration into practice and poor organisational support are some of the specific barriers to physicians’ concordance with CAP guidelines. A number of intervention strategies, such as the implementation of an educational program [12, 13], use of computerised decision support [12], implementation of a clinical pathway [14] and feedback on concordance rate [15], have all been shown to improve physicians’ concordance with CAP guidelines. A systematic review of CAP guideline implementation strategies has recommended using face-to-face education delivery of guidelines, engaging opinion leaders, and providing audit and feedback to prescribers as the most effective strategies to implement CAP guidelines[11].

A 2005 national study assessing physicians’ concordance with CAP guidelines in Australian hospitals, including the Royal Hobart Hospital (RHH, and the hospital in this study), found a low level of concordance (18%) [8]. This finding is similar the 16.1% concordance rate reported in a more recent study at the RHH [16]. A survey [16] and a qualitative study [17] identified specific barriers to physicians’ concordance with the CAP guidelines at the RHH, including the presence of conflicting guidelines, difficulties in calculating the pneumonia severity score, the influence of senior doctors and lack of guideline’s awareness. Considering the findings of these studies, a quality initiative was implemented at the hospital. The initiative had two distinct interventions; a general education intervention and an emergency department (ED) focused intervention.

The aim of this study was to compare the relative effectiveness of the general education and targeted ED focused intervention on improving physicians’ concordance with the national CAP guidelines.

## Methods

This study was conducted at the Royal Hobart Hospital (RHH), the principal referral and teaching hospital in Tasmania, Australia; with 550 beds serving a population of around 240,000. All general medical (n = 120) and ED doctors (n = 40) who were involved in the management of CAP were targeted in our interventions.

The general education intervention involved the development and implementation of a local CAP guideline, based on the national antibiotic guidelines (Therapeutic Guidelines, Antibiotic Version 14, 2010 [5]). The local guidelines were developed collaboratively with the key stakeholders, including representatives from Respiratory, Infectious Diseases, General Medicine, and Emergency Departments during November-December 2012. The guideline was further endorsed by the hospital executive and subsequently published on the hospital intranet in the second week of December, 2012. An email was sent to all RHH medical staff advising them of the release of the local CAP guidelines, which was also supported with educational sessions. Four presentations and six group discussions were held during the second week of December 2012, and two presentations and five group discussions were conducted during the first week of February 2013. All educational sessions were led by the research team, with full support from the hospital’s antimicrobial stewardship team. Wall posters, with the guidelines’ recommendations and key messages, were displayed in all RHH medical departments. Furthermore, a lanyard card (summarising the CAP guideline recommendations) and a pneumonia severity assessment tool were distributed during the educational meetings.

During the ED focused intervention (May to November 2013), a one-page CAP clinical pathway (S1 File) based on the local CAP guidelines was developed and made available in the ED. The CAP clinical pathway was designed to provide ED staff with clear information on appropriate empirical antibiotic therapy for CAP. Two educational sessions were performed with the ED nursing staff to promote the use of the CAP clinical pathway during the second week of May 2013. Furthermore, an email was sent to all medical and nursing staff in the ED to advise them of the presence of the CAP clinical pathway. This was followed by monthly feedback (S2 File) on adherence to the CAP guidelines. The feedback, with detailed explanations, was reported by the primary investigator and sent to the hospital’s antibacterial stewardship team for their review and validation. The feedback was then sent from the stewardship team to the head of the ED department for onward distribution to the ED medical staff.

A list of all patients (≥ 18 years old) who were diagnosed with pneumonia and lower respiratory tract infection were obtained from the RHH’s medical record department and then assessed for eligibility. CAP was identified if a diagnosis of pneumonia was documented in the medical notes or the presence of chest X-ray change was indicated by the physician or the radiologist within 24 hours of presentation. The following exclusion criteria were applied: previous admission to a hospital within the 14 days prior to the admission, aged-care facility resident, immunosuppression [18], taking a corticosteroid, immunosuppressive agent [19], or chemotherapy, history of cystic fibrosis or bronchiectasis, incomplete medical record, and those patients who did not receive antibiotic therapy.

The extracted information from medical records included patients’ demographics and clinical characteristics; chest X-ray, clinical and diagnostic findings; and prescribed antibiotic regimens. The severity of CAP was assessed against the national antibiotic guidelines; specific criteria are shown in Table 1. The empirical antibiotic regimen was considered concordant if the drug, route and dose were all as per the guideline recommendations. Furthermore, only the first administered regimen was assessed to measure guideline adherence.

**Table 1:** Severity assessment of CAP based on Antibiotic Guidelines [5].

Mild CAP	Absence of all of the following: • Respiratory rate > 30 breath per minute • Systolic blood pressure < 90 mm Hg • Oxygen saturation < 92% • Acute onset confusion • Multilobar chest x-ray change • Arterial pH < 7.35 • Partial pressure of oxygen < 60 mm Hg
Moderate CAP	Presence of any of the above criteria
Severe CAP	SMART-COP score ≥ 5 or CORB score ≥ 2

SMART-COP is an acronym for systolic blood pressure, multilobar involvement, albumin, respiratory rate, tachycardia, confusion, oxygen level, and pH (arterial) [20]; CORB is an acronym for confusion, oxygen level, respiratory rate and blood pressure level (systolic and diastolic) [21].

Data were collected on a monthly basis for the period between July 2012 and November 2013, providing five data points pre-intervention, five data points during the general education intervention phase and seven data points during the ED focused intervention phase. The monthly percentages of guideline concordance was computed by dividing the number of patients who received guideline concordant regimen with the total number of patients reviewed in a given month. These data were plotted as a chart to visualise the adherence rates over the study period.

The mean, standard deviation (SD) and standard error of the mean (SEM) for adherence rates at baseline, and during the general and ED focused interventions were calculated. Differences in concordance between study periods were assessed using a one-way analysis of variance (ANOVA) test. If statistical significance was detected, a post hoc analysis was carried out to examine any significant differences across individual time periods.

Patients’ characteristics and clinical outcomes were also examined for the three time periods. The association between categorical data was examined using the Chi-square test. Scaled data was examined using the Kruskal-Wallis test. Statistical analyses were performed using SPSS version 19 (IBM, Armonk, NY, USA). Significance was agreed at two-tail values of p < 0.05.

Ethical approval was granted from the Tasmania Health and Medical Human Research Ethics Committee (THMHREC) (Approval number: H0012810). Data was collected retrospectively following the discharge of patients from the hospital. A waiver of consent request was made, and subsequently approved, by the THMHREC.

## Results

A total of 593 patients were assessed for eligibility, with 195 (32.9%) patients being excluded. The reasons of exclusion were recent hospital admission (n = 54), immunosuppressed patients (n = 86), aged-care facility residents (n = 39), no antibiotic prescribed within 24 hours following admission (n = 7), bronchiectasis (n = 6), cystic fibrosis (n = 1), and incomplete patient’s file (n = 2). The remaining 398 patients were considered as eligible for the study.

Over the entire study period (July 2012 to November 2013), the average number of eligible patients per month was 23. This rate varied during the different phases of the study, with an average of 26 per month for the baseline period (July to November 2012), 18 per month during the general education intervention (December 2012 to April 2013) and 25 per month during the ED focused intervention (May to November 2013).

Table 2 summarises patients’ demographics, length of stay, Charlson comorbidity index [22], chest X-ray change, prior utilisation of antibiotics, severity distribution, documented penicillin allergy, and in-hospital mortality rates among the three phases. There were no significant differences between the three groups on these variables.

**Table 2:** Patients’ demographics and characteristics.

.Study variables	Baseline period (n = 130)	General education intervention (n = 90)	ED focused intervention (n = 178)
**Median age in years**	68	70.5	69.5
**Male (%)**	81 (62.3)	54 (60)	100 (56.2)
**Median length of stay (range)**	3 (1–20)	4 (1–66)	3 (1–57)
**Median Charlson comorbidity Index score (range)**	4 (0–12)	5 (0–11)	4 (0–12)
**Change in chest X-ray noted (%)**	92 (70.7)	62 (68.9)	113 (63.5)
**Severity**			
**Mild (%)**	40 (30.8)	29 (32.2)	49 (27.5)
**Moderate (%)**	57 (43.8)	32 (35.6)	70 (39.3)
**Severe (%)**	33 (25.4)	29 (32.2)	59 (33.1)
**Documented penicillin allergy (%)**	20 (15.4)	13 (14.4)	24 (13.5)
**Prior antibiotics; within 7 days before admission (%)**	22 (16.9)	13 (14.4)	32 (18)
**In-hospital mortality (%)**	7 (5.4)	4 (4.4)	5 (2.8)

During the general education intervention, 39 out of 40 ED medical staff attended the educational sessions; of these, 21 were of a junior grade (interns and residents). The number of doctors who attended from the other medical departments was estimated at 50 (41%). The lanyard card, with the guidelines’ recommendations for the empirical management of CAP, was given to all general medical (n = 120) and ED doctors (n = 40).

**Fig 1:**
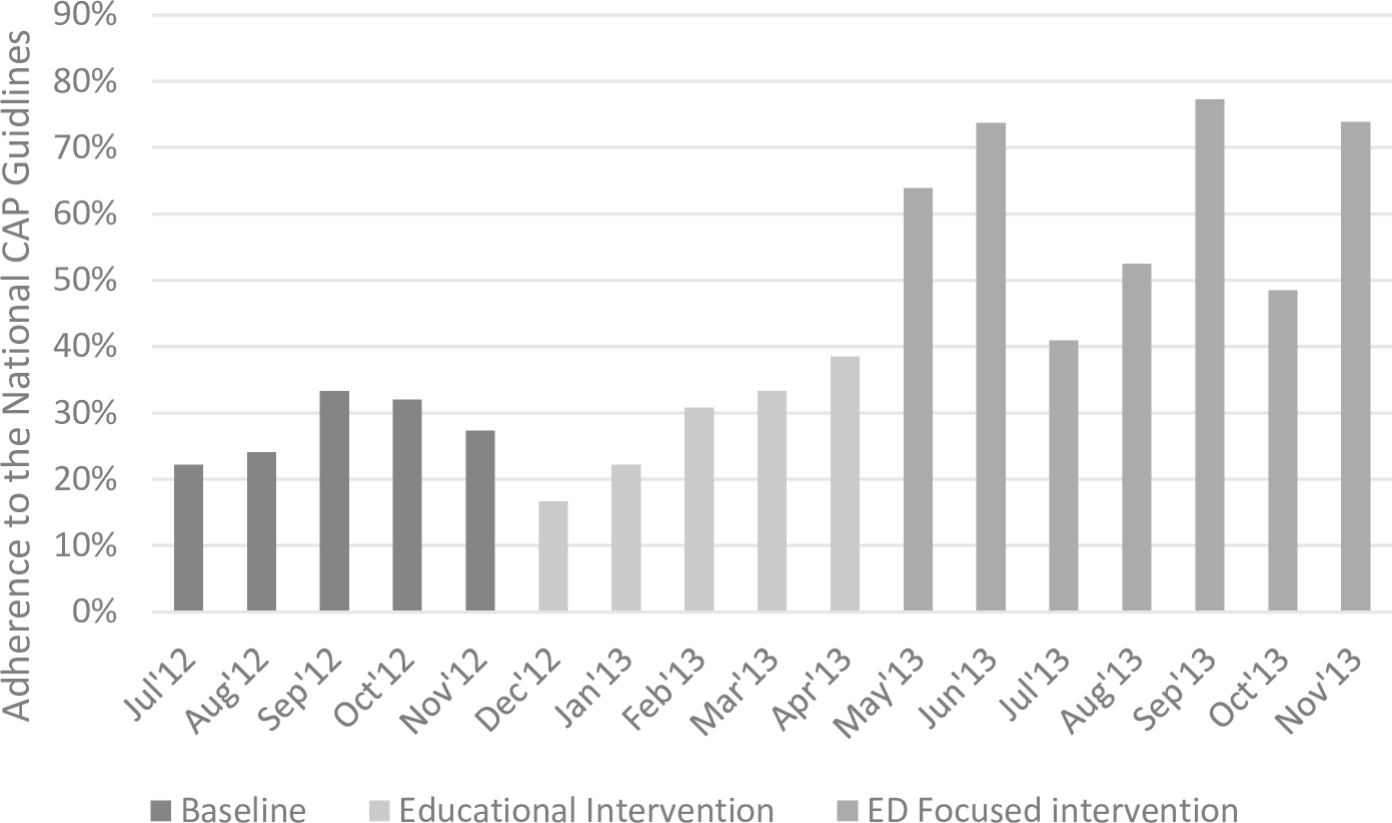
Impact of the General Education and ED Focused intervention on the physicians’ adherence to the national CAP guidelines. Fig 1 summarises the adherence rates to the national CAP guidelines recommendations for the empirical management of CAP between July 2012 and November 2013. During this period, two interventions were implemented. General education intervention that was implemented during mid-December, 2012 to mid-February 2013 with follow-up period till the end of April, 2013 and ED focused intervention that includes a clinical pathway implemented from May 2013, supplemented by monthly audit and feedback (last feedback provided on October, 2013).

As can be seen in Fig 1, adherence rates increased during the ED-focused intervention. Before the general education intervention, the adherence rate began at 22.2% in July 2012. During the ED focused intervention, adherence rates fluctuated between 40.9% and 77.3%. However, all the data points were above any data points in the baseline and general education intervention periods.

The mean adherence percentage for baseline was 28.1 (SEM ± 1.82; SD ± 4.1), general education intervention 31.2 (SEM ± 3.4; SD ± 6.8), and ED focused intervention 61.5 (SEM ± 5.42; SD ± 14.3). One-way ANOVA showed that there was a highly statistically significant difference (p<0.001). A post hoc analysis showed that the adherence rate significantly increased after the ED focused intervention when compared to the baseline period(p< 0.001) and general education intervention (p<0.001). Meanwhile, the general education intervention did not have a significant effect on the adherence rate when compared to the baseline period (p = 0.626).

## Discussion

Community-acquired pneumonia (CAP) is a resource-intensive illness with high mortality in hospitalised patients [1, 2]. Guideline-concordant care has been shown to reduce cost and mortality associated with CAP [6, 7]. Despite the availability of national antibiotic guidelines to treat common infectious diseases, including CAP, physicians’ concordance to CAP guidelines has been poor in Australia [8, 9]. The present study implemented a quality initiative based on the findings of a baseline audit, a survey-based study [16] and a qualitative interview study [17] to improve physicians’ concordance with the Australian CAP guidelines.

One of the recommended strategies for guideline implementation, which has been considered essential to enhance adherence, is the development and implementation of local guidelines that are broadly consistent with the latest version of the national guidelines, with the involvement of key local opinion leaders [23, 24]. This involvement occurred from the earliest stages of development of the local CAP guidelines in our study and continued through to their final approval and implementation. Despite achieving hospital-wide consensus on the CAP guidelines and providing the recommendations at the point of care using lanyard cards, physicians’ concordance to the guidelines remained suboptimal. This is consistent with previously published studies, which have that found educational interventions alone have a limited impact [25, 26].

Physicians’ concordance with CAP guidelines significantly improved following the targeted ED focused intervention that delivered a concise version of the CAP guidelines in the form of a clinical pathway supplemented by a regular audit and feedback cycle. One of the important barriers to physicians’ concordance with practice guidelines is poor clinical workflow integration [10, 11]. Additionally, two earlier studies at the same hospital identified the lack of time and the complexity of calculating a severity score to assess patients’ severity of pneumonia [16, 17]. Providing a clinical pathway at the time of decision making addressed these two important barriers and encouraged guidelines concordance.

There was considerable fluctuation in concordance rates following the ED focused intervention (Fig 1), with troughs coinciding with the commencement of the new three-month junior doctor rotations (Apr-Jun, and Jul-Sep, 2013). The study hospital was a teaching hospital, and most teaching hospitals have clinical rotations for junior medical staff. Adjustment to the new ward’s rules and expectations at the beginning of the rotation might be challenging for junior doctors [27]. The fact that the concordance rate improved soon after the start of the clinical rotation further endorsed the usefulness of implementing an easy to understand pathway tool to improve physicians’ concordance with CAP guidelines.

Provision of audit and feedback to the physicians was found to be an effective component of the ED focused intervention. It is important to note that general reminders and feedback often result in little or no improvement in clinical practice [28]. On the other hand, specific feedback with relevant examples of practice delivered on a regular time interval by a figure of authority may substantially improve clinical practice [29]. Clinicians are often bombarded with alerts for trivial reasons [30], and an equally important consideration in the audit and feedback cycle is to achieve the correct balance regarding detail and quantity of feedback. In our study, the selected patients’ profiles were reviewed and reported by the hospital antimicrobial stewardship team on a monthly basis and were sent to the head of the ED department for distribution to the ED doctors. This frequency of feedback is also supported by the study of Hysong et al. [31], where they found providing feedback less often than once a month rendered it ineffective regarding influencing guideline concordance.

Some limitations of this study should be acknowledged. This was a retrospective study that measured adherence to the empiric choice of antibiotics as per the national CAP guidelines. Other aspects of guidelines recommendations such as de-escalation of antibiotics during the admission, supportive care such as oxygen supplementation and duration of antibiotic treatment were not measured in this study. The definition of CAP used in this study was based on physician’s assessment and/or changes in chest radiograph and may have overestimated the incidence of CAP. Nevertheless, the study aimed to assess the effectiveness of the behavioural change in real-life clinical practice where the diagnosis of CAP is often made on a clinical discretion. The duration of follow-up after each intervention was limited and non-uniform and it is not known if the superiority of the ED focused intervention would have sustained in a longer follow-up period. As mentioned earlier, the study was assessing the relative impact of each intervention in a real-life clinical practice and therefore, we have to rely on a shorter period to sustain clinicians’ interest in this quality improvement project. We excluded patients from aged care facilities; this decision was by the local guideline development group, based on international guidelines from the Infectious Diseases Society of America/American Thoracic Society (IDSA/ATS) who do differentiate between patients living in the community and those who are residents of NH or long care facilities [32], although the national guidelines on CAP do not differentiate between these groups. Despite being a single centre study, the studied hospital represents a typical Australian teaching hospital and the intervention was based on the National Antibiotic Guidelines [5] thus increasing the generalisability of the intervention to other similar hospitals.

## Conclusion

An ED focused intervention, including a CAP clinical pathway and monthly feedback, was a successful strategy to improve adherence to CAP guidelines. In contrast, relying on an educational intervention alone led to a marginal non-significant improvement in adherence. For CAP management, our findings suggest that focusing the intervention at the ED with a clear direction of the desired prescribing practices via a clinical CAP pathway, combined with regular feedback, is a preferable approach.

## Supporting Information

S1 File Community acquired pneumonia pathway tool. Contains a copy of the Community-acquired Pneumonia pathway tool.
(PDF)

S2 File An example of monthly audit and feedback. Contains an example of monthly audit and feedback provided to the physicians at the study hospital.
(PDF)
