# Supplementary material for: Clinical Pathway and Monthly Feedback Improve Adherence to Antibiotic Guideline Recommendations for Community-Acquired Pneumonia
Source: PLoS One. 2016 Jul 25;11(7):e0159467. doi: 10.1371/journal.pone.0159467 (PMC4959693; doi:10.1371/journal.pone.0159467)
Supplement: S1 File — Contains a copy of the Community-acquired Pneumonia pathway tool. (PDF) [file pone.0159467.s001.pdf]

ADULT COMMUNITY ACQUIRED PNEUMONIA (CAP) MANAGEMENT

Tasmania

THO-South

Facility: ____________________

TRIAL FORM

PT ID									

SURNAME…………………………………………… D.O.B……………………

OTHER NAMES……………………………………………………………… SEX:

ADDRESS ………………………………………………………………. MARITAL STATUS:

……………………………………………………………………….. REL :

Attach Patient Sticker Label

FT188520e

Diagnosis of pneumonia: Signs and symptoms consistent with an acute lower respiratory tract infection which may or may not include fever, rigors, cough, sputum production or if chronic cough change in sputum colour, shortness of breath or pleuritic pain AND new or worsening radiographic changes for which there is no other explanation.

CLINICAL ASSESSMENT USING CORB SCORE

Signs/Symptoms (CORB)		Score ONE (1) point for each feature present
**C**onfusion	new onset or worsening of existing state if cognitive impairment present	
**O**xygen	PaO2 60mmHg or less OR Oxygen saturation 90% or less on room air	
**R**espiratory Rate	30 breaths or more per minute	
**B**lood Pressure	Systolic Blood Pressure 90mmHg or less OR Diastolic Blood Pressure 60mmHg or less	
	Total Score:	

RECOMMENDED ANTIMICROBIAL THERAPY (circle selected option and chart on the NIMC)

Criterion	First line therapy	Mild Penicillin Allergy	Severe Penicillin Allergy
**Mild**<br>CORB = 0<br>*Stable comorbidities*	Amoxicillin 1 gram orally 8-hourly<br>OR if atypical pathogens are suspected treat as mild penicillin allergy	Doxycycline 200mg stat then 100mg 12 hourly<br>(If not tolerated then Clarithromycin 500mg 12 hourly)	
**Moderate**<br>CORB = 1<br>*(Assessment of co-morbidities as may require ICU assessment)*	Benzylpenicillin 1.2 gram IV 6 hourly<br>AND<br>Doxycycline 200mg orally stat then 100mg orally twice daily<br><br>(Or if Doxycycline not tolerated then use Clarithromycin 500mg orally 12-hourly)	Ceftriaxone 1 gram IV daily<br>AND<br>Doxycycline 200mg orally stat then 100mg orally twice daily<br><br>(Or if Doxycycline not tolerated then use Clarithromycin 500mg orally twice daily)	Moxifloxacin 400mg orally daily
**Severe**<br>CORB = 2 or more<br>*(Consider ICU Consultation)*	Ceftriaxone 1 gram IV daily<br>AND<br>Azithromycin 500mg IV daily	Ceftriaxone 1 gram IV daily<br>AND<br>Azithromycin 500mg IV daily	Moxifloxacin 400mg IV or orally daily AND Azithromycin 500mg IV daily

INVESTIGATIONS *MUST NOT* DELAY ANTIMICROBIAL THERAPY.
INVESTIGATIONS TO BE PERFORMED INCLUDE:

- ☐ Full Blood Examination, electrolytes, urea and creatinine
- ☐ Chest X-ray

ADDITIONAL INVESTIGATIONS FOR MODERATE-SEVERE PNEUMONIA:

- ☐ Sputum microscopy, culture and sensitivity (M,C,S)
- ☐ Arterial blood gases in patients with severe pneumonia or at risk of hypercapnic respiratory failure
- ☐ Urinary Antigens (pneumococcal/*Legionella*)
- ☐ Blood cultures
- ☐ Other testing may include: Respiratory PCR Testing OR other testing as per RHH Adult CAP Guideline on intranet.

Legend

IV - Intravenous
PaO2 – partial pressure of oxygen
mg - milligram
mmHg–millimetres of mercury
PCR – polymerase chain reaction

Print Name:	Designation:
Signature:	Date:

ADULT CAP MANAGEMENT

TIME TO ANTIBIOTICS IS PARAMOUNT. ANTIBIOTIC ADMINISTRATION WITHIN 4 HOURS OF ARRIVAL IS ASSOCIATED WITH DECREASED MORTALITY AND LENGTH OF STAY.

Penicillin Hypersensitivity/Severe life-threatening penicillin allergy

Severe life-threatening penicillin allergy, or Type 1 hypersensitivity, are Immunoglobulin E mediated reactions resulting in the release of histamines and other mediators from mast cells and basophils. Reactions occur immediately to one hour after exposure, and are characterised by urticaria, angioedema, bronchospasm and anaphylaxis.

Discussion with Respiratory Team for admission is recommended in:

- Moderate or Severe pneumonia
- Mulitlobar pneumonia
- Significant parapneumonic effusion
- Patients with pneumonia who are well known to respiratory team.

Indications for referral to the intensive care team include:

- CORB score 2 or more (severe pneumonia)
- Severe/refractory hypoxemia (fraction of inspired oxygen requirement 0.4 or above)
- Septic shock
- Multi-organ failure
- Marked agitation/delirium
- Loss of airway protection
- Complex co-morbidities

'Atypical' pneumonia

Atypical pneumonia includes: *Chlamydophilia/Chlamydia* species, *Mycoplasma pneumoniae* and *Legionella* species).

Serum for atypical serology (*Chlamydophilia/Chlamydiai* species, *Mycoplasma pneumoniae* and *Legionella* species) will only be tested with the receipt of convalescent serum in the laboratory (taken at 2-4 weeks post illness) or in discussion with the Medical Microbiologist.

Less common but important aetiology:

Legionella diagnosis has important public health implications. Consider and ensure testing if concern especially if renal failure and/or gastrointestinal symptoms are present.

PLEASE REFER TO THE MANAGEMENT OF ADULTS WITH COMMUNITY ACQUIRED PNEUMONIA GUIDELINE ON THE INTRANET FOR THE COMPLETE GUIDELINE.
