# Supplementary material for: Clinical Pathway and Monthly Feedback Improve Adherence to Antibiotic Guideline Recommendations for Community-Acquired Pneumonia
Source: PLoS One. 2016 Jul 25;11(7):e0159467. doi: 10.1371/journal.pone.0159467 (PMC4959693; doi:10.1371/journal.pone.0159467)
Supplement: S2 File — Contains an example of monthly audit and feedback provided to the physicians at the study hospital. (PDF) [file pone.0159467.s002.pdf]

S2. Example of the feedback provided to the Physicians at the Royal Hobart Hospital

Case#[a]	Use of Pathway	Severity of Pneumonia noted	Severity based on CORB[b]	Antibiotic regimen	Guideline concordant	Chest X Ray changes	Comments
1	Yes	Yes	Severe	Ceftriaxone 1 g IV and Azithromycin 500 mg IV	Yes	No	
2	Yes	Yes	Severe	Ceftriaxone 1 g IV and Azithromycin 500 mg IV	Yes	No	Penicillin allergy
3	Yes	Yes	Mild	Amoxycillin 1 g PO and Doxycycline	No	No	
4	Yes	Yes	Mild	Amoxycillin 1 g PO	Yes	No	
5	No	Yes	Mild	Benzylpenicillin 1.2 g IV	No	No	
6	No	No	Mild	Ceftriaxone 1 g IV and Doxycycline 200 mg PO	No	Yes	
7	No	No	Mild	Amoxycillin 1 g IV	No	No	Discordant route
8	Yes	Yes	Moderate	Ceftriaxone 1 g IV and Doxycycline 200 mg PO	Yes	Yes	Penicillin allergy
9	No	No	Severe	Ceftriaxone 1 g IV and Azithromycin 500 mg IV	Yes	Yes	
10	No	No	Mild	Amoxycillin 1 g PO	Yes	No	

[a] Actual patient record’s number is replaced with a case# to protect patients privacy

[b] CORB is an acronym for confusion, oxygen level, respiratory rate and blood pressure level (systolic and diastolic)
